# Durability of response to intra-articular corticosteroid injections with triamcinolone hexacetonide in juvenile idiopathic arthritis

**DOI:** 10.1186/1546-0096-10-S1-A47

**Published:** 2012-07-13

**Authors:** Jaya Srinivasan, Themba L Nyirenda, Kathleen A Haines, Yukiko Kimura, Suzanne C Li, Jennifer E Weiss

**Affiliations:** 1 Hackensack University Medical Center, Hackensack, NJ, USA; 2 University of Medicine and Dentistry of New Jersey, Newark, NJ, USA

## Purpose

Intra-articular corticosteroid injection (IACI) with triamcinolone hexacetonide (TH) is a mainstay of therapy for patients with juvenile idiopathic arthritis (JIA). Our aim was to determine factors that affected the durability of response to IACI with TH in patients with JIA.

## Methods

A retrospective chart review was conducted of all JIA patients who received IACI from 6/05 to 3/10, and had at least six months of follow-up. Data collected included demographic information, JIA subtype, date of injection and arthritis flare, type of joint injected and concomitant medications. Any joint that did not flare by the study end date in 9/10 was censored. Time to flare of arthritis was calculated based on the Kaplan-Meier product limit estimator. Two-sided log-rank test was conducted to compare the time to flare within each characteristic group: joints, diagnosis, medications. All analysis in this study was performed using SAS 9.2 (SAS Institute Inc, Cary, NC).

## Results

There were 112 patients (83 females) and 198 separate joints included in the study. Fourteen (7.1%) joints did not respond to their first IACI. Of the 184 joints that did respond to the initial IACI, 99 (53.8%) fully improved and did not relapse during the study period (duration of follow-up: mean 723.7 ±421 days; median 553 days, IQR 385-994). There was a significant difference in time to flare between elbow and wrist joints. Although hip joints showed the shortest time to flare, the sample size was too small to tell if the difference was significant.

**Table 1:** Factors affecting flare in JIA patients post-ICAI (n=184)

Characteristic	Flared (5)	Time to flare median (days)	p-value**
**Joint**			
Knee (n=112	55 (49.1)	972.0	
Ankle (n=30)	12 (39.9)	903	0.0297†
Wrist (n=17)	11 (64.7)	569.0	
Elbow (m=14)	2 (14.3)	243	

**Diagnosis**			
Oligoarthritis (n=97)	39 (40.2)	1042.0	
Polyarthritis (n=40)	15 (37.5)	903.0	
Extended oligoarthritis (n=23)	14 (60.9)	569.0	0.0003††
Systemic arthritis (n=14)	11 (78.6)	276.0	
Enthesits-related arthritis (n=6)	3 (50.0)	629.0	
Psoriatic arthritis (n=2)	1 (50.0)	797.0*	

**Medications**			
DMARD			
No (n=116)	53 (45.7)	846.0	0.8660
Yes (n=68)	32 (47.1)	903.0	

**Biologics**			
No (n=141)	64 (44.4)	972.0	0.0729
Yes (n=43)	21 (48.8)	615.0	

**NSAID**			
No (n=84)	42 (50.0)	797.0	0.1353
Yes (n=100)	43 (43.0)	972.0	

*Median not estimated by SAS; the reported value is the 25^th^ percentile.

**p-value<0.05 was considered statistically significant.

†A significant difference between joint types was found for elbow vs. wriset joint (p=0.0180) after adjusting for multiple testing using a Hochberg procedure.

††A significant difference between JIA subtypes was found for oligoarthritis vs. systemic arthritis (p=0.0015) and between polyarthritis vs. systemic arthritis (p=0.0084) after adjusting for multiple testing using Hochberg procedure.

Twenty to 36% of each joint type received a second IACI. About a third of the re-injected knee joints required a third IACI, and all four of the re-injected hip joints received a third IACI.

**Table 2:** Repeat IACI in JIA patients with arthritis flare

Joint	Flare afer second IACI n(%)		Flare after third IACI n(%)	
	Total injected	Joints flared	Total injected	Joints flared

Knee	37	15 (40.54)	13	6 (46.15)
Ankle	6	6 (100.00)	0	0 (0.00)
Wrist	5	1 (20.00)	0	0 (0.00)
Elbow	3	1 (33.33)	1	1 (100.00)
Hip	4	4 (100.00)	4	4 (100.00)
Total	55	27	18	11

## Conclusion

IACI is an effective therapy for patients with JIA with the majority of patients having complete and long-lasting response to IACI. Over half of the injected joints did not relapse after a mean and median follow up of 23.7 months and 18.2 months, respectively. There was a significant difference in median time to flare between the elbow and wrist joints. Knees had the longest median time to relapse and hips the shortest; however, a larger sample is needed to determine if these represent significant differences. Systemic arthritis showed the shortest time to relapse, and was statistically different from oligoarthritis and polyarthritis. Concomitant medications did not have a significant effect on flare times. A larger study population is needed to better evaluate the effect of joint type and other factors on risk of recurrent flares.

## Disclosure

Jaya Srinivasan: None; Themba L. Nyirenda: None; Kathleen A. Haines: None; Yukiko Kimura: None; Suzanne C. Li: None; Jennifer E. Weiss: None.

